# Health-Resorts in the West of England and South Wales

**Published:** 1890-12

**Authors:** E. J. Slade-King

**Affiliations:** Medical Officer of Health for Ilfracombe


### ILFRACOMBE.

BY

E. J. SLADE-KING, M.D., D.P.H.

*Medical Officer of Health for Ilfracombe.*

---

FOR the last fifteen years North Devon has been gaining in popularity as a health-resort, and the number of people who are recommended there in search of health increases year by year, not only in summer, but in winter-tide also. Since the two direct lines of railway—the Great Western and London and South Western—by their accelerated train service have made Ilfracombe so accessible from London, this is the point which most visitors select as their head-quarters when visiting North Devon. Ilfracombe so little resembles other watering-places both in its position and surroundings, that an adequate description is a somewhat difficult task, and it requires to be visited by those who would fully realise how unlike it is to almost all other coast towns.

It is most picturesquely situated on the southern shore of the Severn Sea. From the North Foreland to Morte Point extends a grand coast-line of steep cliffs, fronted by jagged rocks, broken only by "mouths," as Lynmouth, Heddonsmouth, Watermouth, Wildersmouth, and some two or three other "mouths," through which the streamlets collected in the grassy Combes have broken the barrier and leaped into the sea. The Combe along which the Wilder stream finds its way is known as Ilfracombe; or, as it indifferently seems to have been spelled, Ilfordscombe, Alfredscombe, or Ilfarcombe. The land-surface is either depressed by four deep valleys—Lee, Slade, Score, and Chambercombe—or so undulated that not a single acre is level enough for a cricket ground; and even the railway terminus is of necessity perched on the spur of a hill, standing abruptly some 200 feet above the level of the streets. The coast-line is fine; and outstanding prominently, Helesborough, the Lantern Hill, Capstone, and the seven peaked "Torrs," form the most lovely walks, and perhaps the best marine promenade in the kingdom. These walks are zigzagged on the face of the hills, and lead by a succession of easy gradients to the summit, whence a glorious view of sea and land may be seen. The greatest want perhaps is in the partial absence of a sand beach: but that want is compensated for in more ways than one; and the children must substitute pebbles for sand in their toy-spades, though the wish for the softest sand can be gratified by a visit to Woollacombe, where there is a clear sandy beach, without let or hindrance. Nowhere are there found such lovely rock terraces, such charming secluded coves, such pretty bathing-places, such beautiful lanes ramifying southward and eastward, down which to catch on each retrospect "the many-twinkling smile of ocean," as at Ilfracombe. Fashion and custom fix the season for August and September, though the mildness of the climate wins not infrequent winter visitors—health seekers longing for pure air, or to escape the severity of the weather in the East and North. But perhaps, of all seasons, early summertide is the most inviting, when the orchards are pink with apple-blossom, the trees and hedgerows are decked in their freshest livery of green, when the tall grass waves gracefully by the goldcups, and thousands of silvery daisies glitter beside the blue speedwells, while the scented honeysuckle and briar-roses are unfolding, and the blue sky overhead is flecked with breezy cloudlets. Autumn also is a pleasant time; it is genial on these western shores, and narrows the limits of winter's icy reign. But here winter can hardly be called icy; there seldom average more than five frosts in the twelve months, some years escaping them altogether, and our meadows are green and our wild flowers blossom in succession all the year round.

*Local Productions.*—The High Street is the business part of the town; the shops are good and supply every necessary want, while to the lovers of lace and china a walk through that same High Street will prove very delightful. The markets are large, moderate, and well supplied. Three local productions are important to the invalid: clotted cream, a pleasant substitute for cod-liver oil; four-year-old Exmoor mutton, which is almost as well-flavoured as venison; and laver *(Porphyra vulgaris* and *Porphyra lancinata)*, a sea-weed which grows on the rocks at low-water mark. It is prepared by the country people for market by being washed in running water for a long time, and then gently simmered over a slow fire for hours together. It is served very hot, mixed with vinegar or Worcester sauce and butter. It contains a small proportion of iodine and bromine, which renders it a most useful adjunct to diet in cases where alterative treatment is desirable.

Milk is abundant and rich. In the season, game and rabbits are plentiful. Black game from Exmoor is occasionally for sale. Fish is abundant, being procured from both the North and South Devon coast, as well as landed by steam trawlers directly from the new fishing grounds north west of Lundy.

There are excellent fishing stations within easy reach of Ilfracombe, for those who like to kill their own dish of trout; and even a salmon is no unusual prize in the Taw or Torridge.

The whole of the local ferns, in which this district is unusually rich, and many others, may be purchased in the market.

*Improvements.*—Artificial means have been used to enhance the beauties which Nature has so lavishly bestowed. A fine Parade has been made round the Capstone: the Victoria Promenade, or the "Shelter," as it is more frequently called, has proved beneficial; for, being situated at the south foot of the Capstone Hill, it is especially suited for a winter walk. It is a building of iron and glass, with the flooring of encaustic tiles, and seated throughout: it is planted with creepers and ornamental shrubs; with a band-stand, where a band plays in severe weather. On cold sunny days the inside of the Shelter enjoys the genial warmth of summer; and invalids can drive there in the useful donkey-chairs, which will put them down close to the entrance, so that they can get in and out without any difficulty, and can sit inside listening to the band, enjoying the warmth and greenery, without fear of taking cold. Admission is free.

*Sanitation.*—The town is drained by extensive public sewers, with which all the houses are connected. These sewers consist of Annerly glazed pipes, are well ventilated, both at their dead ends and along their course, by lofty air shafts. They have two outfalls, extended by iron pipes into the tideway, where the deep, strong current, running four knots an hour and rising 35 feet in this Channel, soon sweeps all sewerage matter out of sight. The town- and house-refuse is daily collected, all organic matters being separated and burnt.

*Water-supply.*—The water-supply is constant, and under high pressure, coming partly from deep springs, and partly from an undulating collecting area lying above and on both sides of the Slade Valley. The watershed is pierced by the great railway-cutting. The collected water is stored in two deep reservoirs; it is then passed on to five beds, where it is filtered through sand and gravel from the Spratt Ridge in the river Taw. It is a soft and excellent water for all domestic purposes.

The following analysis of Ilfracombe water may interest some of our readers:

| | Grains per Imperial Gallon |
|---|---|
| Sulphate of Lime ... ... ... ... ... | 1.522 |
| Sulphate of Magnesia... ... ... ... ... | .385 |
| Nitrate of Magnesia ... ... ... ... | .712 |
| Chloride of Magnesia ... ... ... ... ... | .918 |
| Chloride of Sodium ... ... ... ... ... | 3.391 |
| Soluble Silica ... ... ... ... ... ... | .050 |
| Oxides of Iron and Alumina ... ... ... | .070 |
| Oxidisable Organic Matter ... ... ... ... | .179 |
| Total Solids ... ... ... Grains | 7.227 |
| Total Hardness ... ... ... ... | 4½ degrees. |
| Permanent Hardness ... ... ... | 4 degrees. |

*Death-rate.*—With such advantages it is not to be wondered at that Ilfracombe is healthy, and its death-rate so low, seldom exceeding 15 per 1000. A large percentage of the population attain the age of 60 years and upwards; and no less than eight inhabitants, during the past half-century, have reached the patriarchal age of 100.

*Vital Statistics.*—The following are the *crude* death- and birth-rates for the last two years:

| | 1888. | | 1889. |
|---|---|---|---|
| A Mortality of Residents and Visitors of | 14.3 | ... | 14.5 |
| A Birth-rate of ... ... ... ... | 25.5 | ... | 25.3 |
| An Infant Mortality per 1000 born of ... | 99.3 | ... | 89.4 |
| A Zymotic Mortality ... ... ... ... | .0 | ... | .7 |

These death-rates are without doubt greater than the true death-rates, which in towns of small size, subject to a large periodical influx of strangers, are of very difficult calculation.*

Ilfracombe is well equipped with an Infectious Diseases Hospital, a Mortuary, a Hospital for Accidental Cases, Fire Brigade, and Life Boat; and can claim to be one of the very few towns which the Government Inspector declared, on his visit during the last cholera scare, to be entirely free from sanitary defects.

*Climate.*—With regard to climate, it may be summarised as essentially oceanic, and, compared with many health-resorts throughout the kingdom, occupies one of the first places in regard to mildness. It is conspicuous for the absence of those extremes so prejudicial to health, especially to invalids. The summers are comparatively cool—thunder and lightning being of rare occurrence. The air during this period is remarkable for frequent agitation: generally bracing, without being keen; fresh, without pungency. Some people object to

* Ilfracombe is a U.S. district co-extensive with the Parish. It covers an area of 5,358 acres; and contains a population of between seven and eight thousand.

On a given week in August the number of strangers sleeping in the town reached nearly 9,000.

Ilfracombe, saying it is often too windy to be exactly pleasant; but these brisk movements of the atmosphere are of the utmost value to the invalid, affording in the hottest days opportunity for restoring the exhausted tone of the system; and these over-fastidious objectors must remember that health must be first considered. The winters are mild and equable, snow seldom falling,—and if it does, never lying on the ground,—and railway and telegraphic communication but seldom interrupted. It is owing to the Gulf Stream that our Ilfracombe winters are so very unlike the same period of the year in an eastern county. The air of an eastern winter irritates the invalid's chest; but here in the west it is "breathing made easy," for the soft air, loaded with sea-moisture, soothes while it penetrates the lungs. Tubercle may remain quiescent for years if all causes of irritation are avoided; and of these none is so common or so unavoidable as the sudden contact of cold, raw air. Then, why has Ilfracombe such a reputation as a sanatorium for those suffering from disease of the kidneys, from gout, from disorders of the liver and stomach? It is in a great degree because the climate induces, by its equality and softness, a regular action of the skin and the various mucous surfaces. Another important fact which must be mentioned is the dryness of the soil: the roads may at times be rough, but they are never muddy in the true sense of the word. The winding lanes, so beautiful with ferns and wild flowers, and the delicious home walks, so special an enjoyment at Ilfracombe, are free from this great drawback to country pleasures. Rarely is it necessary for the most delicate to be homebound: to be detained indoors from wet underfoot is almost unknown, as the surface of the soil on the cessation of rain at once becomes dry.

*Progress.*—Comparatively few years ago Ilfracombe was but a small village, known to few, and visited by still fewer. During the past fifteen years it has made rapid progress, and the last two or three years have so increased its size that our once distant parish church has nearly become the centre of the town, and the wild Torrs are on their land side studded with modern villas, and their bold sea-fronts are carved with pathways easy of access to adventurous donkey-chairs. How is it there has been such rapid progress? Ilfracombe boasts of no trade or manufacture; the people are employed in agriculture, coasting and fishing, in retail trade, or lodging-house keeping.* But one thing it has which has brought it into repute, and that is the giving of health. It is for that reason it has become so noted. At a disadvantage when compared with many watering-places which are situated near large centres of industry, on account of its isolation, still it holds its own with Llandudno, Rhyl, Southport, and Blackpool (places so dear to the dwellers in the Black Country), as the number of summer visitors proves; and the steady increase of winter trade shows that as a health-resort it is becoming worthily famous. Here we breathe only the purest air. If, on the one hand, it is a disadvantage to be far away from our big manufacturing cities, on the other hand it has its important advantages. Turning from the fresh sea-breezes, and getting away from the town, we have the sweet, bracing air which blows on us from hill and fell, from miles of open country stretching far away on all sides, filling our lungs with the needful oxygen, and

* The old saying "Everyone to his trade" certainly stands good of Ilfracombe. Lodging-letting is the trade, and is most excellently provided for; and if strangers will only avoid August, there is no English town where life may be lived in lodgings so well and so moderately as in 'Combe.

giving to our weary frames new health and life. Moreover, Ilfracombe and the adjacent country possess sufficient character and sufficient originality—if we may so use the word—of atmosphere and scenery, as to render a resort to North Devon, next to special continental travel, the most desirable thing in the world for the seeker of direct change. This change is now gained over a direct route, suited for the most delicate sufferer, by a journey which will cause the slightest possible amount of fatigue, and the length of which, if necessary, can be agreeably broken. Thus, we cannot wonder that, possessing such splendid scenery, such advantages of soil and situation, Ilfracombe should have made such advances, and from being merely a creek, sheltering pilots and channel coasters, and largely suspected of smuggling, it has now justly become so favourite a resort.

*Dress.*—The conventional rules which govern dress are here greatly relaxed; and provided the toilette is graceful and easy, its fashion will scarcely bar the visitor from mixing at any hour with the gay throngs that crowd the Shelter, promenade on the Capstone, gaze on the players in the public tennis-courts, or sit in thick rows on the rocks to watch the doings of the wonderful and unrivalled "Punch," who, from being a mere bird of passage, has been tempted to make Ilfracombe his permanent home. Here grave citizens, who pursue their daily avocations in black frock-coats and high hats, rush recklessly into straws, many-coloured caps, striped flannel jackets of brilliant hue—locally known as "Blazers,"—demonstrating how much the pleasure of seaside life is enhanced by the near approach to the freedom and conditions of uncivilised society.

In conclusion, it may be affirmed that the clear, mild climate of Ilfracombe, the well-established reputation of which has of late been submitted to such severe scrutiny, is suited for the most delicate invalid, and has rather increased than diminished in celebrity, for its curative agencies rest on a reasonable and satisfactory hypothesis. Still, it is no panacea for disease, and, like other permanent remedies, it is slow and gradual in its action on the human frame. True it is that persons remove here after partial recovery from acute illness, and find often that the action of the air is rapid in hastening convalescence; but in more chronic cases the invalid must be warned against impatience, remembering the gradual inroad of disease, the slow departure from the normal limits of health, and feel sure, however favourable may be the result, still that a few short weeks cannot in reason suffice for retracing the uphill pathway of health. The health-seeker, however, may be assured that, leaving the result with the Almighty Disposer of events, he adopts in selecting Ilfracombe as a sanatorium a most effectual means for the restoration of his debilitated frame. The vast increase of our urban populations, and the necessarily artificial lives they live, render their yearly outing a necessity as well as a pleasure; and a better playground than the Ilfracombe district it would be hard to find. The air, so breezy and free from organic matters; the genial yet tonic climate, and the dry soil, which permits of the maximum of out-door exercise; the equality of temperature; the absence of thunder and lightning, which are so disastrous to sufferers from epileptic fits; the sparseness of population; the very small proportion of old houses, old courts, and old streets; the lofty and eye-striking frontage which forms the seaboard of the town; the vast expanse of open and almost uninhabited country which extends for miles inland, rising with gentle gradients to an altitude of over a thousand feet, a grassy land broken by rounded hilltops, torrs, and combes, and accessible in all parts by lanes and field-paths; the view seaward, where the eye watches with changeful interest the vast trade of the Bristol Channel passing and repassing, thus avoiding the depressing monotony of sameness; the perfect drainage; the abundance and purity of the water; the ease and amplitude of sea and land excursions, which reach Clovelly, Lynton, Bideford, Exmoor, and Dartmoor; the simple pleasures and early hours which fashion the lives of most of the visitors to 'Combe, may not, indeed, be sufficient inducement to allure the devotees of fashion or the frivolous idler, but are all-important to the genuine seeker of health, who will here recognise a glorious temple where Nature needs no priest of Æsculapius to interpret her ritual, but, face to face with her votaries, freely bestows large gifts of health and healing.

